# On the Completeness of Existing RNA Fragment Structures

**DOI:** 10.1093/gpbjnl/qzaf127

**Published:** 2025-12-18

**Authors:** Xu Hong, Jian Zhan, Yaoqi Zhou

**Affiliations:** Institute of Systems and Physical Biology, Shenzhen Bay Laboratory, Shenzhen 518055, China; Institute of Systems and Physical Biology, Shenzhen Bay Laboratory, Shenzhen 518055, China; Ribopeutic Inc, Guangzhou International Bio Island, Guangzhou 510320, China; Institute of Systems and Physical Biology, Shenzhen Bay Laboratory, Shenzhen 518055, China

**Keywords:** RNA fragment, RNA modeling, RNA structure, RNA pseudo-torsion angle, RNA reference frame

## Abstract

The success of protein structure prediction by the deep learning method AlphaFold 2 naturally raises the question of whether similar success can be achieved for RNA structure prediction. One reason for the success in protein structure prediction is that the structural space of proteins, from the fragment level to the domain level, has been nearly complete for many years. Here, we examined the completeness of RNA fragment structural space at the di-, tri-, tetra-, and penta-nucleotide levels. We show that the number of non-redundant structural fragments at the tetra- and penta-nucleotide levels is in the midst of exponential increase, suggesting that the structural space currently observed in RNA is far from complete. Thus, more concerted efforts are clearly needed to improve the speed and methods of experimental determination of RNA structures to go beyond the limited structural space observed in RNAs. Moreover, the reference frame composed of three sugar-ring atoms near the base side (O4′, C1′, and C2′) exhibits the least structural diversity among existing RNA structures, suggesting it as the most stable platform for building other parts of RNA structures.

## Graphical Abstract

**Figure:**
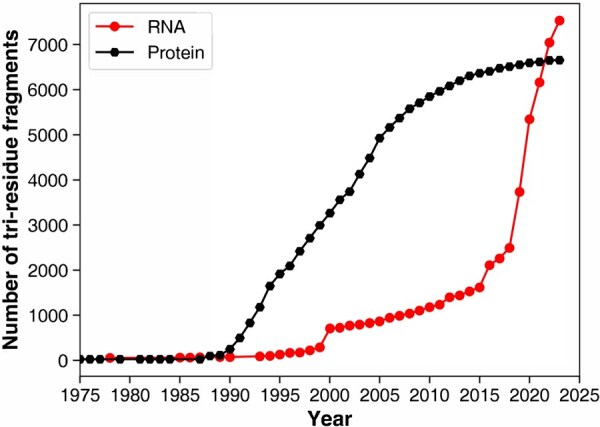

## Introduction

The recent success of AlphaFold 2 [1] for protein structure prediction relies on the large database of protein structures (∼ 200,000) in the Protein Data Bank (PDB) [2]. This dataset has long been comprehensive, achieving nearly complete structural coverage at the 3- and 4-residue fragment levels [3], 7- and 9-residue fragment levels [4], and even the structural domain level [5]. Such extensive, near-complete structural data enabled the dominance of fragment-based assembly methods for *de novo* protein structure prediction before 2018. Furthermore, they also laid the groundwork for the success of end-to-end deep learning techniques, such as AlphaFold 2, in achieving high-accuracy predictions [6].

Like proteins, many RNAs have unique three-dimensional (3D) structures that are largely determined by their sequences. These structured RNAs (*e.g.*, tRNA, rRNA, ribozymes, riboswitches, and long non-coding RNAs) are key to elucidating their diverse functional mechanisms [7]. However, unlike proteins, RNA structures pose greater challenges for determination by nuclear magnetic resonance spectroscopy, X-ray crystallography, or cryogenic electron microscopy due to their unique physicochemical properties [7]. Currently, RNA-containing structures account for only 3% of all structures deposited in PDB [2]. This raises the question of whether the current fragment-structural library contained in PDB is sufficient for artificial intelligence (AI)-based learning [8–13] or fragment-based assembly for RNA structure prediction. Fragment-based techniques have been employed for predicting RNA 3D structures (*e.g.*, Rosetta [14], SWM [15], FARFAR [16], FARFAR2 [17], 3dRNA [18–21], FebRNA [22]), RNA–RNA complex structures [23], and protein–RNA complex structures [24].

Previously, RNA structures were commonly characterized by six torsion angles in the sugar-phosphate backbone. The distribution of these torsion angles is often restricted [25,26], particularly for those rotated around C5′–C4′ and C1′–N9/N1 bonds [27,28]. To simplify the representation, Keating *et al.* introduced two pseudo-torsion angles: θ (P_i_, C4′_i_, P_i+1_, and C4′_i+1_) and η (C4′_i-1_, P_i_, C4′_i_, and P_i+1_) [29,30]. However, the conformations of two fragments could differ drastically even if they fall within the same pseudo-torsion bin (*e.g.*, a typically employed 5-degree bin) [31]. Thus, relying on these two pseudo-torsion angles may underestimate the structural diversity of RNAs.

**Figure 1:**
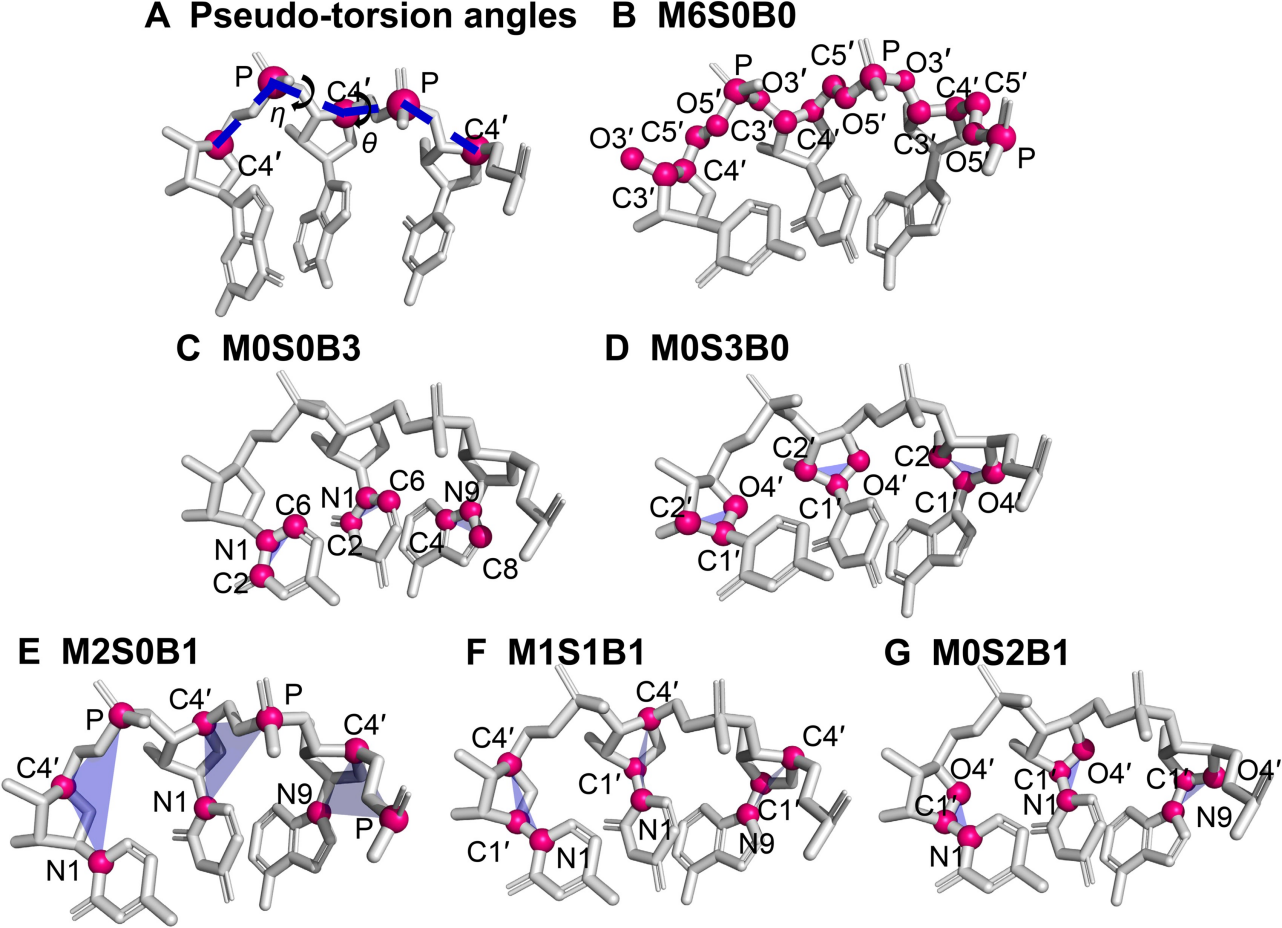
Frames used to represent the nucleotides **A**. Two pseudo-torsion angles (*θ*, *η*) for RNA backbone structure characterization are rotated about the P_i_-C4′_i_ and C4′_i_-P_i+1_ pseudo-bonds, respectively. Specific atoms used in the M6S0B0 (**B**), M0S0B3 (**C**), M0S3B0 (**D**), M2S0B1 (**E**), M1S1B1 (**F**), and M0S2B1 (**G**) reference frames, where M, S, and B denote the numbers of backbone, sugar, and base atoms, respectively. The blue triangle represents the frame under different representations.

The completeness of a fragment structure library for proteins is typically measured according to the structural similarity between fragment backbone atoms, because protein structures are characterized by regular secondary structure elements (helices and sheets) with side chains acting as rotamers around main chains [32]. RNA structures, on the other hand, are driven by base pairing of nucleotide bases, whereas the backbone is treated as rotamers around bases [33,34]. Consequently, both backbone and base atoms should be taken into account for the structural characterization of RNA fragments. Clearly, using different combinations of backbone and base atoms as reference frames for structural comparison might yield different results on structure-space completeness. Thus, we investigated several reduced representations (mainchain backbone only, base only, sugar only, and mixed, as shown in **Figure 1**) and compared the structural spaces characterized by these representations. We show that an RNA structure represented by three atoms in the sugar ring near the base side has the least conformational diversity (*i.e.*, sugar is the most stable part of RNA structures), when compared to conformations represented by mainchain backbone or sidechain bases, whereas two mainchain atoms (C4′ and P) together with one base atom (N1 for C/U or N9 for A/G) are sufficient to represent the entire RNA structure, as structural differences characterized by this reference frame show a near-perfect correlation to those based on all heavy atoms in RNA.

## Results

### Fragments with similar pseudo-torsion angles can have dissimilar structures

In previous studies, pseudo-torsion angles (*θ* and *η*) were employed to classify all fragment nucleotide conformers [29,30]. If tri-nucleotide conformations are classified according to 5° bins for *η* and *θ*, nearly complete coverage of all possible grid points has been achieved since 2022 (5176/5184 in 2022 and 5178/5184 in 2023), as shown in Figure S1. However, two fragments with nearly identical pseudo-torsion angles do not necessarily have the same structure. As shown in Figure S2, there is a moderate correlation [Pearson’s correlation coefficient (PCC) = 0.4] between the full-atom root-mean-square distance (RMSD) values and the distance of two pseudo-torsion angles (the definition of angle distance is described in Materials and methods). Moreover, 0.6% of pairwise structures with an angle distance of < 10° have RMSD > 1 Å. This is a small percentage, confirming the usefulness of the two pseudo-torsion angles to represent the overall RNA structure. However, this percentage is non-negligible, as we shall see that the fraction of new tri-nucleotide fragments is dominated by the structures with Δ*η* < 5° and Δ*θ* < 5° but ΔRMSD > 1 Å from old structures.

**Figure 2:**
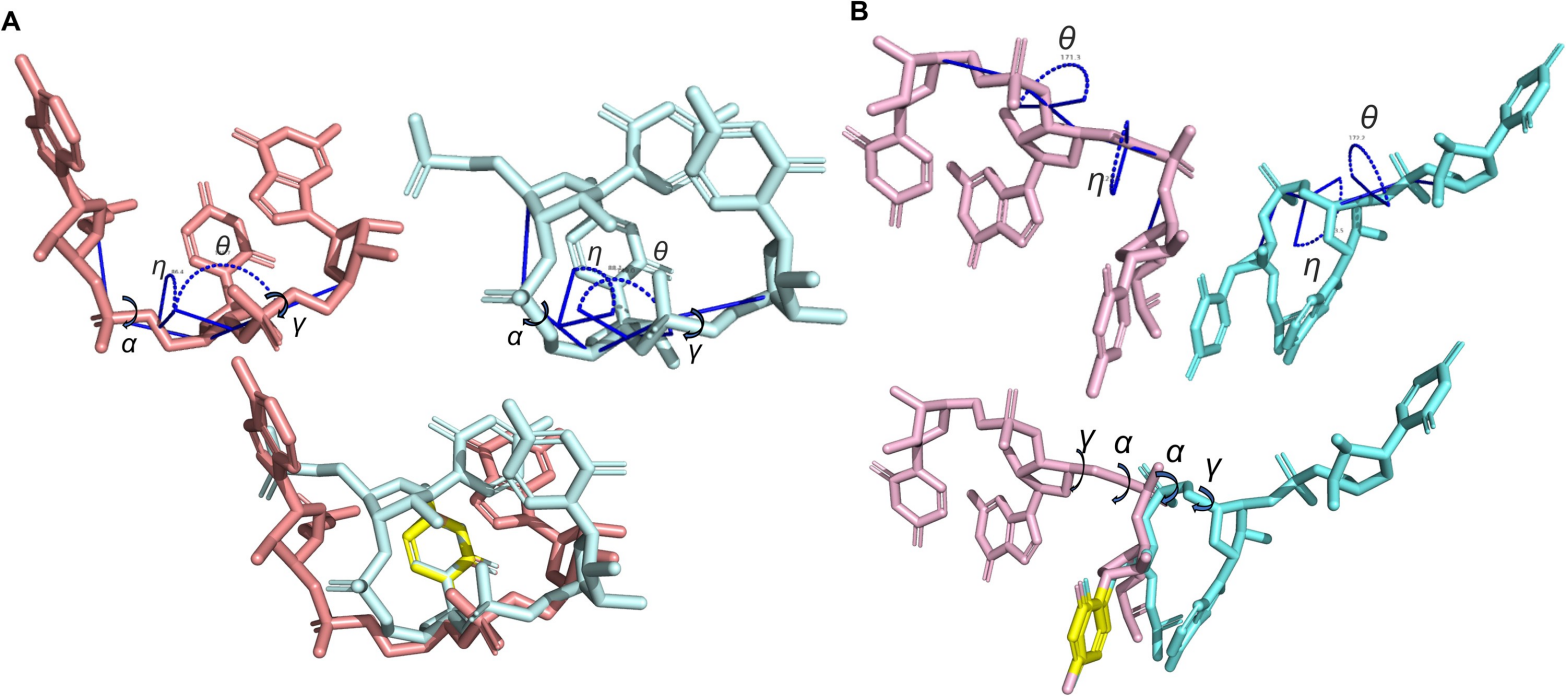
Two examples that tri-nucleotide conformers share similar pseudo-torsion angles but have large structural differences The structural alignment was based on the aromatic rings shown in yellow. **A**. Structural comparison between the fragment of nucleotides 17–19 of a riboswitch (2kxm_A, C3′-endo sugar pucker, in pink) and the fragment of nucleotides 2581–2583 of a ribosome (7pwo_1, C3′-endo sugar pucker, in blue). **B**. Structural comparison between the fragment of nucleotides 42–44 of a synthetic RNA (7pdu_B for C3′-endo, in pink) and the fragment of nucleotides 3150–3152 of a ribosome (8euy_1 for C2′-endo, in blue).

Two such examples are illustrated in **Figure 2**. In Figure 2A, two tri-nucleotide structures, which were derived from the nucleotides 17−19 of a riboswitch (2kxm_A) and 2581–2583 of a ribosome (7pwo_1), respectively, have nearly the same pseudo-torsion angles (*η* angle: 86.4° *vs*. 88.1°; *θ* angle: −145.7° *vs*. −149°). However, the RMSD values between these two fragments are 3.55 Å for the M0S0B3 base atom representation (Figure 1C) and 2.44 Å for the M6S0B0 mainchain atom representation (Figure 1B). These two fragments would be considered as different structural fragments based on our 1 Å threshold. One reason for this large RMSD difference is that the differences between the backbone γ torsion angles about the C5_2_′–C4_2_′ bond (59.6° *vs*. −29.5°) and the α torsion angles about the P_3_–O5_3_′ bond (−80.9° *vs*. 84.0°) of the two conformers are nearly 90° and 160°, respectively. Similarly, in Figure 2B, the structures of nucleotides 42–44 of a synthetic RNA (7pdu_B for C3′-endo) and nucleotides 3150−3152 of a ribosome (8euy_1 for C2′-endo) share nearly identical pseudo-torsion angles (*η* angle: 122.5° *vs*. 123.5°; *θ* angle: 171.3° *vs*. 172.2). However, the RMSD values between these fragments for M0S0B3 base frame and M6S0B0 mainchain frame are 3.13 Å and 2.96 Å, respectively. This can be partially explained by the fact that the differences between α angles about the P_2_–O5_2_′ and the γ angles about the C5_2_′–C4_2_′ of the two conformers are near 170° (α angle: 174.3° *vs*. −20.7°; γ angle: −176.7° *vs*. 13.6°).

### Comparison between reduced representations

**Figure 3:**
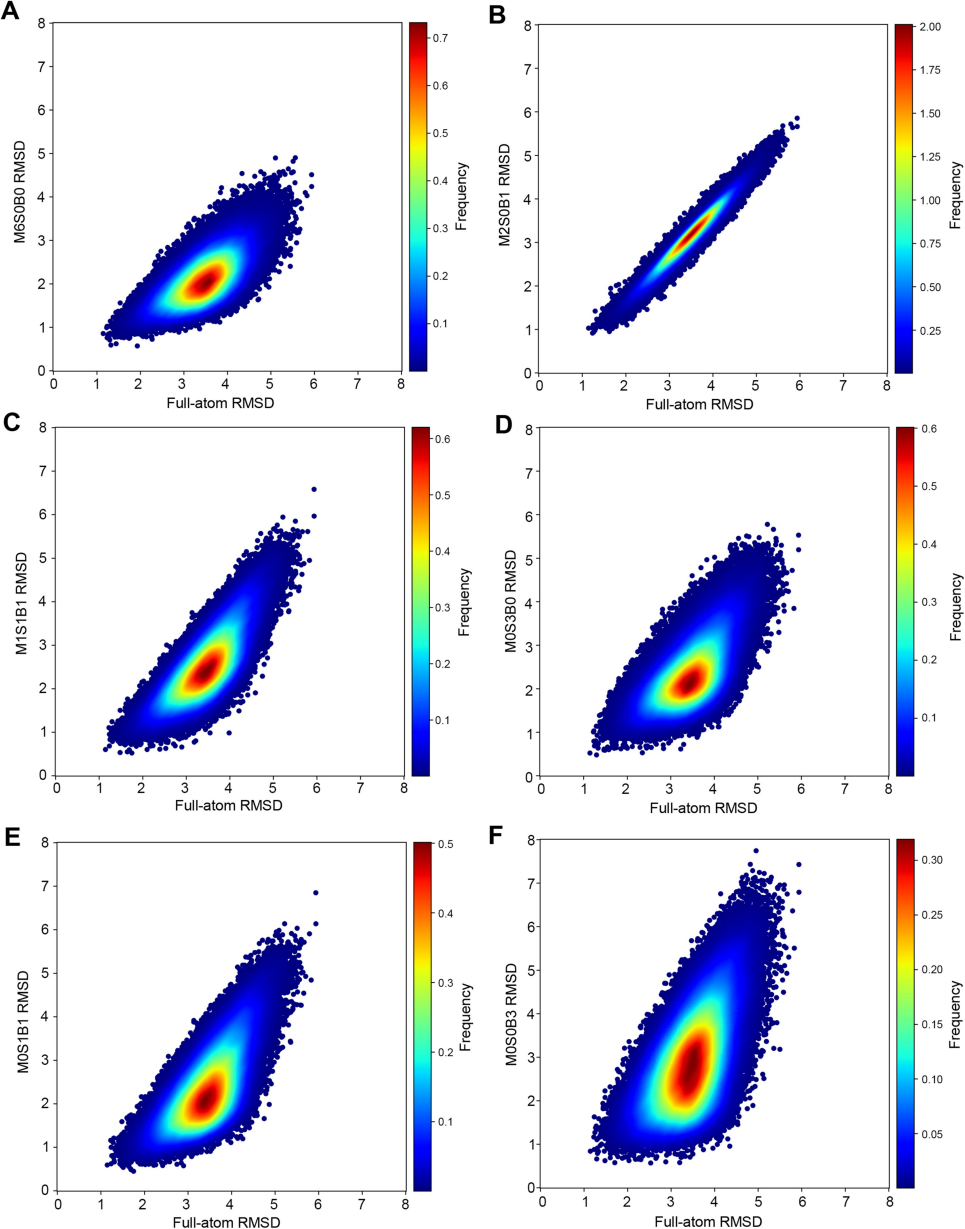
Correlation between all-atom RMSD values and those from other representations **A**. Mainchain backbone frame (M6S0B0). **B**. Mainchain-base-mixed frame (M2S0B1). **C**. Mainchain-sugar-base-mixed frame (M1S1B1). **D**. Sugar ring near the base side (M0S3B0). **E**. Sugar-base-mixed frame (M0S2B1). **F**. Base frame (M0S0B3). The horizontal and vertical axes indicate full-atom RMSD and frame RMSD, respectively. Frequency refers to the density of occurrences of points in the area. RMSD, root mean square distance.

We assessed whether a reduced representation is a good approximation to the full-atom representation by examining the correlation between RMSD values from the reduced representation and those from the full-atom representation. We employed 7840 representative tri-nucleotide fragments clustered on the basis of all common heavy atoms from structures available before 2018, and obtained the RMSD values for all studied representations. We compared the RMSD values using all common heavy atoms (shown on x-axis in **Figure 3**) to those using simpler reference frames (shown on y-axis in Figure 3). Here, we employed density plots, in which regions are colored from blue to dark red, indicating progressively higher point frequencies. These plots are based on 100,000 points randomly selected from all pairwise alignments among 7534 M2S0B1 representative tri-nucleotide fragments.

As shown in Figure 3, the highest PCC of 0.950 was observed between the RMSD value of the M2S0B1 frame and that of the full-atom representation. PCC values ranged from 0.678 between full-atom and M0S0B3 base representations, 0.718 between full-atom and M6S0B0 mainchain representations, 0.731 between full-atom and M0S3B0 sugar representations, 0.753 between full-atom and M0S2B1 representations, and up to 0.821 between full-atom and M1S1B1 representations. Thus, while base pairing drives RNA folding, the best representation for the whole RNA structure is two atoms on the mainchain backbone (C4′ and P) and one atom at the base (N1 for C/U or N9 for A/G). This is somewhat expected, as this representation includes the mainchain backbone as well as the structure furthest away from the backbone (nucleotide base).

### Clustering of RNA fragments

**Figure 4:**
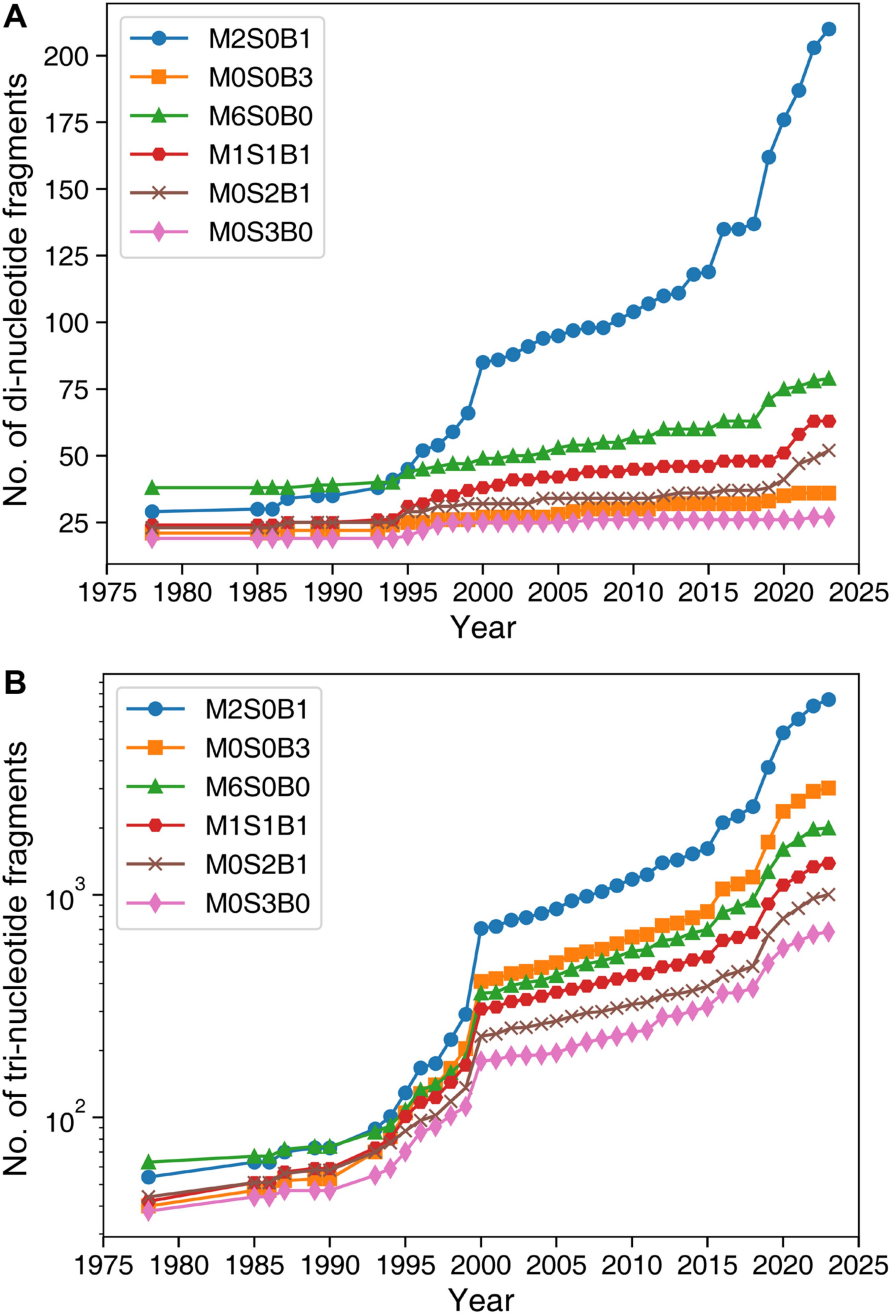
Growth of RNA structural fragments over time **A**. Di-nucleotide fragments. **B**. Tri-nucleotide fragments. The cumulative counts are shown for six reduced structural representations (M6S0B0, M2S0B1, M1S1B1, M0S3B0, M0S2B1, and M0S0B3).

All fragments were clustered based on frame RMSD values. Non-redundant fragments refer to representative fragments from different clusters that were separated by more than 1 Å RMSD. **Figure 4**A and B showed the cumulative number of non-redundant di- and tri-nucleotide fragments over the year for different representations, respectively. The results showed that the fragment structures based on three atoms of the sugar ring near the base side (M0S3B0) had the least number of non-redundant structural fragments (*N*_M0S3B0_ = 27 for di-nucleotides and *N*_M0S3B0_ = 681 for tri-nucleotides) among all compared representations. An interesting observation was that the fewer the reference atoms in the sugar ring, the greater the number of structural fragments (*N*_M0S3B0_ < *N*_M0S2B1_ < *N*_M1S1B1_), confirming that the sugar ring is the most stable part of the RNA structure. Another interesting observation was that the number of fragment structures of the mainchain backbone was less than that of the base reference frame (*N*_M6S0B0_ < *N*_M0S0B3_), indicating that the relative positions between bases can have more structural variation than the backbone. The M2S0B1 representation exhibited the largest number of structural fragments (*N*_M2S0B1_ = 210 for di-nucleotides and *N*_M2S0B1_ = 7534 for tri-nucleotides), consistent with its superior performance in characterizing the overall RNA structure based on RMSD correlations (Figure 3). Interestingly, the trend of the number of fragment structures for base representation was opposite for di- and tri-nucleotide fragments. For tri-nucleotides, the number of fragments in the base representation (*N*_M0S0B3_) was larger than in other representations except M2S0B1 (*N*_M0S0B3_ > *N*_M6S0B0_ > *N*_M1S1B1_ > *N*_M0S2B1_ > *N*_M0S3B0_). In contrast, for di-nucleotides, the N_M0S0B3_ count was lower than in M6S0B0, M1S1B1, and M0S2B1 representations. A possible explanation is that local di-nucleotide restrictions in mainchain and sugar structures led to a limited possibility for structural presentation of base atoms. This phenomenon could also be observed for tetranucleotides. In this case, the fragment count for the M0S0B3 representation was lower than for M2S0B1, but higher than other representations, as discussed below. Figure 4 also showed that the increment of di- and tri-nucleotide fragments continued for N_M2S0B1_, while the increment of di- and tri-nucleotide fragments for other representations seemed to plateau. For example, the slopes for all representations except M2S0B1 were below 0.05, while the slope for the M2S0B1 representation exceeded 0.08.

We examined 486 tri-nucleotide fragments of M2S0B1 representation newly added in 2023. We found that 479 fragments (98.6%) had Δ*η* < 5° and Δ*θ* < 5°, but ΔRMSD > 1 Å compared with structures available prior to 2023. This finding confirms the limitation of characterizing RNA structure space by two pseudo-torsion angles *η* and *θ* alone.

We observed sudden increases in the number of RNA fragments in the years 2000, 2019, and 2020. The first high-resolution complete atomic structure of the large ribosomal subunit (1ffk) contributed to the jump in 2000 [35]. Two new ribosome structures (70S ribosome complex with erythromycin (6s0x) [36] and 50S ribosome bound to compound 40e (6pcs) [37] were responsible for most of the new structural fragments in 2019. Another ribosome structure (yeast 80S ribosome complex, 6woo) [38] in 2020 led to a substantial rise in new fragment structures. Thus, the plateaued increment in RNA fragments for some representations at the di- and tri-nucleotide levels might be disrupted if a new, previously unseen RNA structure type is found.

**Figure 5:**
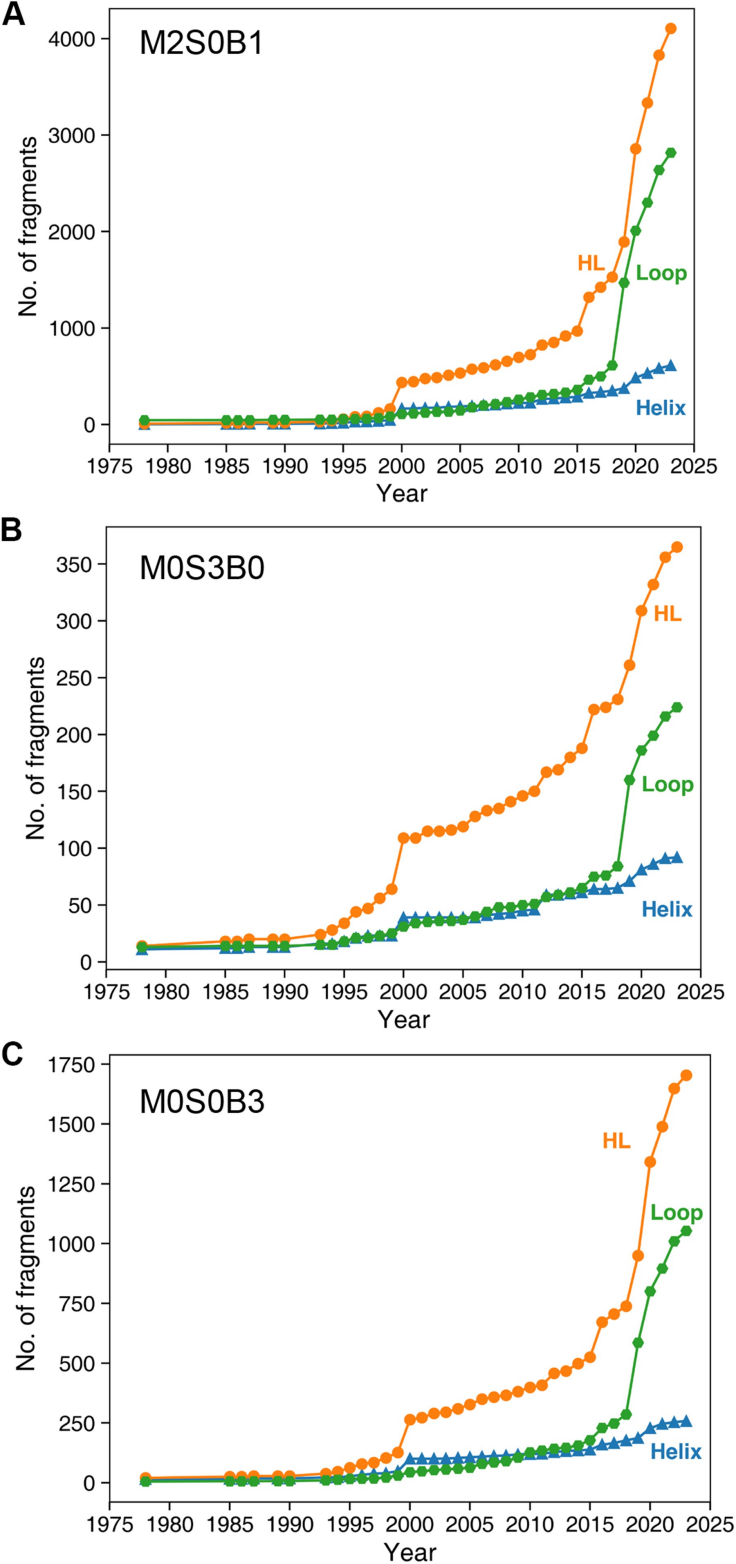
The cumulative number of tri-nucleotide fragments located in helix, loop, and HL regions as a function of the year **A**. The M2S0B1 representation. **B**. The M0S3B0 representation. **C**. The M0S0B3 representation. The base pairs used to identify helix, loop, and mixed helix-loop regions were annotated by DSSR [49]. DSSR, dissecting the spatial structure of RNA. HL, helix-loop.

For tri-nucleotides, we further analyzed all representative conformers according to whether they are located in helical regions (where all nucleotides are base-paired), loop regions (where no nucleotides are base-paired), and mixed helix-loop regions that contain both base-paired and unpaired nucleotides. As shown in **Figure 5**, regardless of representation, the number of tri-nucleotide fragments was the highest in mixed helix-loop (HL) regions (*e.g.*, ∼ 4105 for M2S0B1 in 2023), followed by the loop regions (*e.g.*, ∼ 2817 for M2S0B1 in 2023), whereas the helical regions had the lowest number of fragments (*e.g.*, ∼ 612 for M2S0B1 in 2023). Thus, the structural space of helical regions for tri-nucleotides was mostly explored as the slope was close to zero for helical regions, slowed down for some representations (M0S0B3 and M0S3B0), but remained rapidly increasing for the mixed helix-loop and loop regions in the M2S0B1 representation. Near completeness in tri-nucleotide helical conformations was consistent with the simpler structural patterns in the helical regions.

**Figure 6:**
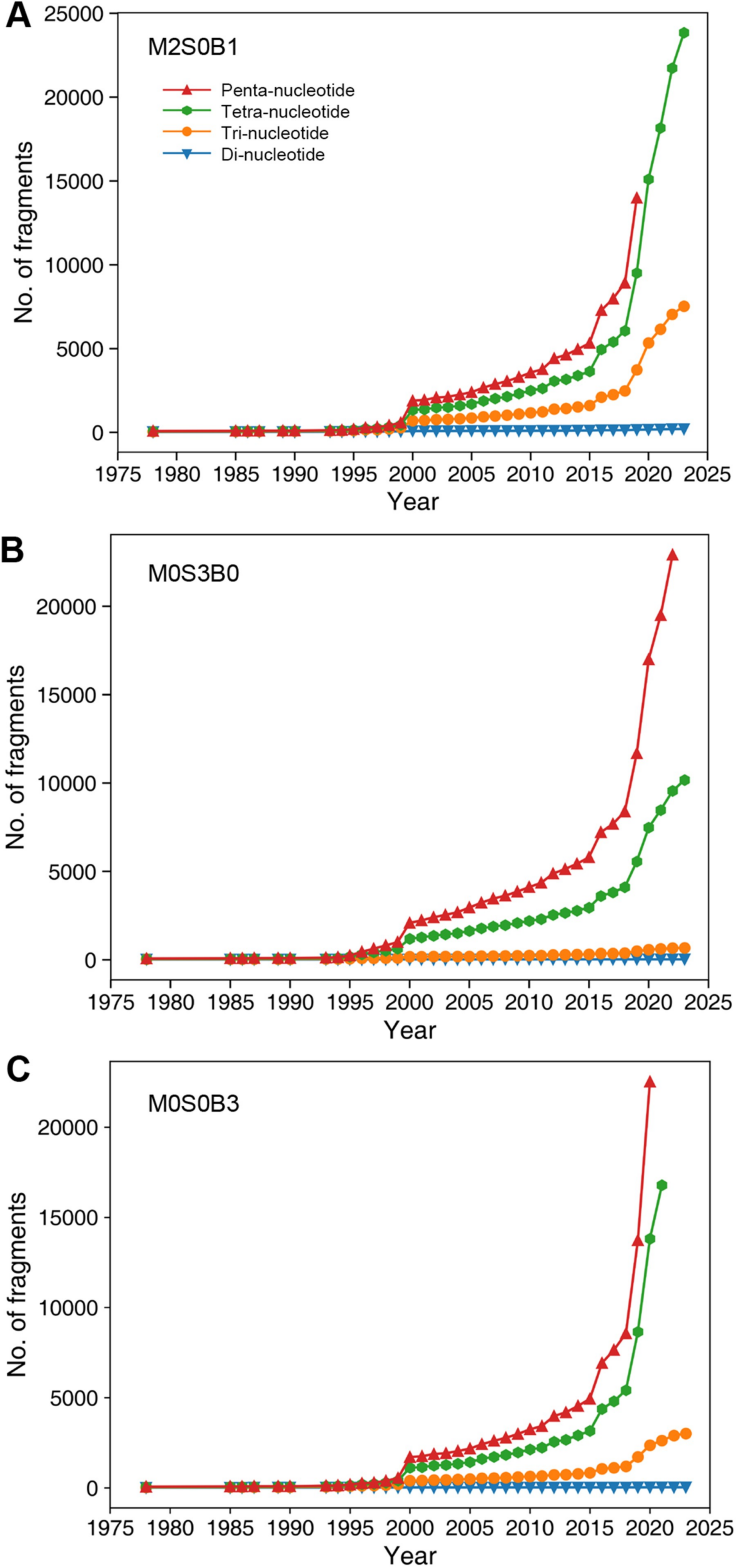
The cumulative number of structural fragments at the level of bi-, tri-, tetra-, and penta-consecutive nucleotides as a function of the year **A**. The M2S0B1 representation. **B**. The M0S3B0 representation. **C**. The M0S0B3 representation.

We further examined tetra- and penta-nucleotide conformers based on the M2S0B1, M0S3B0, and M0S0B3 frames. The trend for the number of these fragments is shown in **Figure 6**. Unlike di- and tri-nucleotide fragments, the numbers of fragments for these three representations increased exponentially, highlighting the limited structural space explored by known RNA structures.

### Classification based on the trinucleotide sequence

Given the highly similar physicochemical properties of bases, one would expect that all sequences could have a similar number of fragments once the structural space of the fragments reached completeness. As shown in Figure S3, the number of fragment structures for the same tri-nucleotide sequence was uneven, ranging from 300 to 800, which served as another indication of incompleteness in fragment structures. Furthermore, even for the two sequences with the most tri-nucleotide structural fragments after clustering (UUU and GAA), the completeness had not yet been reached, as shown in Figure S4.

## Discussion

To understand why predicting RNA structures remains highly challenging, we examined the fragment structures of RNAs at the di-, tri-, tetra-, and penta-nucleotide levels. By clustering these structures according to the RMSD values, we found that the number of tetra- and penta-nucleotide fragments increases exponentially, while the increase in di- and tri-nucleotide fragments was plateaued for some representations but not for the M2S0B1 representation. It is clear that the structural space of RNAs observed so far is far from complete.

One might argue that there is a slowdown in the growth of new fragment structures, at least at the di- and tri-nucleotide levels for some representations. This slowdown may be temporary. New structural space can be expanded rapidly whenever a new structure is solved, as occurred around the years 2000, 2019, and 2020 (Figure 4). Thus, we may observe another significant expansion if the structure for a new class of RNA molecules, such as long non-coding RNAs, is solved in the future.

The results described above are certainly dependent on the threshold employed for defining new structures (1 Å RMSD employed here). Obviously, a lower RMSD threshold will lead to more clusters, whereas a higher RMSD threshold will lead to fewer clusters, as shown in Figure S5. However, the qualitative trend will remain the same regardless of the actual RMSD threshold employed. Here, we chose 1 Å RMSD because this threshold has been used previously for proteins [39] and RNAs [24].

How to increase the chances of finding more new structural fragments? We examined the relationship between newly identified fragments and the number of RNA structures in non-redundant sequences deposited in PDB each year. There were 877 high-resolution RNA chains after removing redundancy by CD-HIT [40] with a sequence identity cutoff at 0.8 until the year 2024. This cutoff was commonly employed by previous studies for sequence clustering [41,42]. As shown in Figure S6, there was a moderate positive correlation (ranging from 0.47 to 0.71) between the number of new structural fragments and the increased number of deposited structures in non-redundant sequences. Thus, solving more structures with non-redundant sequences is an effective way to expand the structural space.

Given the incompleteness of RNA fragment structures, it is unlikely that the success of AlphaFold 2 in high-accuracy protein structure prediction can be reproduced for RNA structure prediction, as AlphaFold 2 benefited from nearly complete space for protein structural fragments [3–5]. Several deep learning methods for RNA structure prediction have been developed. They include end-to-end techniques such as AlphaFold 3 [10], Rhofold+ [12], and Nufold [13], as well as combinations of deep learning with energy-based folding, such as trRosettaRNA [9], RoseTTAFoldNA [11], and DRfold [8]. However, the performance of AlphaFold 3 is even less accurate on RNA targets in Critical Assessment of Structure Prediction techniques (CASP 15, 2022) than that from Alchemy-RNA2 [43], which is based on the BRiQ statistical energy function for folding [33]. The results from CASP 16 (2024) further confirmed that both AI and non-AI based techniques are unable to predict unseen structures: the more dissimilar the target structures are from known structures, the lower the prediction accuracy becomes (https://predictioncenter.org/casp16/doc/presentations/). Thus, expansion of RNA structural space as well as improving algorithms for deep learning and/or energy functions are clearly required for further advancement in RNA structure prediction.

Different representations were employed previously for RNA structure prediction by deep learning. The M2S0B1, M1S1B1, and M0S3B0 representations were employed in DRfold [8], RhoFold [12], and Nufold [13], respectively. Our results, after analyzing 6 representations, indicate that the M2S0B1 representation can better characterize the overall structure of an RNA, given its RMSD values having a near-perfect correlation with the full-atom RMSD (Figure 3). However, the M2S0B1 representation has the highest number of structural fragments, whereas M0S3B0 has the lowest number of structural fragments, which is even much lower than the number from the pure backbone (M6S0B0) or the pure base representation (M0S0B3). This suggests that the M0S3B0 frame has the least structural variation and is therefore the most structurally stable. Thus, the M0S3B0 frame may be the best for model building for deep learning algorithms or fragment-based assembly.

This work was limited to the structural space of the continuous nucleotide fragments. Although we did analyze the structural space of continuous fragments in helical regions and found that the increase in structural fragments is significantly slower in helical regions than in loop and mixed regions, the structural space of base-pair stacks remains to be investigated, which would involve non-continuous fragments and non-sequential structural alignment [44,45]. Previously, base pairs were classified according to the edges utilized in hydrogen bonding (Watson–Crick edge, Hoogsteen edge, and sugar edge) [46,47]. It would be interesting to examine how these edges play a role in different structural patterns of base-pair stacking [48]. We hope to address this question in future studies.

## Materials and methods

### Structure datasets

We downloaded all RNA-containing structures from PDB [2] as of Mar 5th, 2024. We extracted structures solved by X-ray diffraction or cryo-electron microscopy at a resolution better than 3 Å. This led to a collection of 6447 RNA chains released before Jan 1st, 2024, for the time-dependent analysis in this work. We further extracted continuous fragments of these structures at the levels of di-, tri-, tetra- and penta-nucleotides after excluding missing residues and modified residues. Time stamps of these structures were recorded to construct time-dependent fragment libraries.

### Clustering of structural fragments

Structural similarity between two continuous fragments was measured by the RMSD between their reference-frame atoms. We examined different reference frames, since they can provide information on structural diversity, including conformations of mainchain backbones and sidechains, as well as the most stable structural frame. We first investigated the characterization of RNA backbones by θ and η, rotational angles around the pseudo-bonds between P_i_ and C4′_i_ and between C4′_i_ and P_i+1_, respectively (Figure 1A) [30]. Then, we further examined several representations of fragment structures: (1) all mainchain backbone atoms (P, O5′, C5′, C4′, C3′, and O3′, denoted as M6S0B0, Figure 1B), (2) key base atoms (N9, C8, and C4 for A and G, N1, C6, and C2 for C and U, denoted as M0S0B3, Figure 1C), (3) key sugar atoms (O4′, C1′, and C2′, denoted as M0S3B0, Figure 1D), (4) a combination of two backbone atoms and one base atom (C4′, P, and N1 for C/U, or C4′, P, and N9 for A/G, denoted as M2S0B1, Figure 1E), (5) a combination of one backbone atom, one sugar atom, and one base atom (C4′, C1′, and N1 for C/U, or C4′, C1′, and N9 for A/G, denoted as M1S1B1, Figure 1F), and (6) a combination of two sugar atoms and one base atom (O4′, C1′, and N1 for C/U, or O4′, C1′, and N9 for A/G, denoted as M0S2B1, Figure 1G). We also obtained RMSD values calculated based on all the heavy atoms common for all bases used for comparison (P, OP1, OP2, O5′, C5′, C4′, O4′, C3′, O3′, C2′, O2′, C1′, N9/N1, C8/C6, and C4/C2).

We followed a previous study on protein fragment structure clustering [3] by calculating RMSD values between the frame atoms of the two continuous fragment structures. All fragments were initially clustered with an RMSD threshold of 0.5 Å to obtain the representative fragment for each structure cluster and calculate the number of fragment structures in each structure cluster. Meanwhile, all representative fragments from all structures in each year were further clustered with an RMSD threshold of 0.5 Å to obtain the representative fragments and the number of fragment structures for each cluster in each year. Then, the representative fragments from each year were compared with all representative fragments from previous years, and the fragments with RMSD > 1 Å were counted as the new structural fragments found in each year. The Kabsch algorithm [50] was utilized for calculating RMSD values. Hierarchical clustering implemented using the SciPy package [51] in Python was employed to cluster all fragments based on their RMSD values. The distance between two newly formed clusters, *u* and *v*, was calculated using the equation below:


(1) $$d(u,v)=\sum_{ij}d(u[i],v[j])/(|u|*|v|)$$

where the summation is over all structures in clusters, |*u|* and |*v|* are the cardinalities of clusters *u* and *v*, respectively. Two RMSD thresholds were used in this study (0.5 Å and 1 Å). An RMSD threshold of 0.5 Å was employed for the initial fine-grained clustering, so that locating new structural fragments at the RMSD threshold of 1 Å could be made on representative structures for the clusters formed at the finer grid, thereby reducing the computational cost. Using 1 Å as the RMSD threshold for defining the minimum distance between new structural fragments and “old” structures was employed previously for RNA tri-nucleotide fragments [24].

We adopted the definition of angle distance from reference [31].


(2) $$\mathrm{Angle}\;\mathrm{distance}=\sqrt{\frac{1}{2}(\Delta \eta^{2}+\Delta \theta^{2})}$$

(3) Δη=min(|η1−η2|,360°−|η1−η2|)

(4) Δθ=min(|θ1−θ2|,360°−|θ1−θ2|)

## CRediT author statement

**Xu Hong:** Data curation, Writing – original draft, Writing – review & editing, Visualization. **Jian Zhan:** Conceptualization, Writing – review & editing, Supervision. **Yaoqi Zhou**: Conceptualization, Resources, Writing – original draft, Writing – review & editing, Supervision, Project administration, Funding acquisition. All authors have read and approved the final manuscript.

## Competing interests

Jian Zhan is a current employee of Ribopeutic Inc. All the other authors have declared no competing interests.

## Supplementary material

Supplementary material is available at *Genomics, Proteomics & Bioinformatics* online (https://doi.org/10.1093/gpbjnl/qzaf127).

## Supplementary Material

qzaf127_Supplementary_Data
