# Supplementary material for: On the Completeness of Existing RNA Fragment Structures
Source: Genomics Proteomics Bioinformatics. 2025 Dec 18;23(6):qzaf127. doi: 10.1093/gpbjnl/qzaf127 (PMC13197129; doi:10.1093/gpbjnl/qzaf127)
Supplement: qzaf127_Supplementary_Data [file qzaf127_supplementary_data.zip › qzaf127-Supplementary Material Captions-sjc-HX.docx]

# **Supplementary material**

**Figure S1** **Coverage of trinucleotide fragments in pseudo-torsion angle space**

The cumulative number of structures in distinct 5° bins in two pseudo-torsion angles (*η* and $\theta$). Since 2022, this count has approached the maximum possible value of 5,184 (72x72), indicating that the trinucleotide fragments nearly cover the *η*-$\theta$ space.

**Figure S2** **Correlation between full-atom RMSD and pseudo-torsion angle distance**

The full-atom RMSD versus the distance between two pseudo-torsion angles ($\sqrt{\frac{1}{2}({\Delta\eta}^{2}+{\Delta\theta}^{2} )}$). This figure contains 100,000 points randomly selected from pairwise alignments of 7,534 representative three-nucleotide fragments. The Pearson’s correlation coefficient between the full-atom RMSD and the distance is 0.40.

**Figure S3** **Sequence-dependent counts of trinucleotide structural fragments**

The number of structural fragments based on different trinucleotide sequences. All structural fragments were clustered based on either M2S0B1, M0S3B0, or M0S0B3 representations as labeled.

**Figure S4** **Growth of structural fragments for sequences UUU and GAA over time**

The number of structural fragments for two trinucleotide sequences (UUU and GAA) clustered by the M2S0B1, M0S0B3, and M0S3B0 representations as a function of the year.

**Figure S5 Dependence of fragment cluster counts on RMSD threshold**

The number of representative RNA fragments at different RMSD cutoffs for trinucleotide (UUU) fragments as labeled.

**Figure S6** **Correlation between newly solved RNA structures and new fragment discovery**

The number of newly added structural fragments at a given year versus the number of deposited RNA structures in non-redundant sequences for the same year. The horizontal and vertical axes indicate newly added non-redundant sequences and newly added non-redundant fragments, respectively. **A.** M6S0B0 representation (Pearson’s *r*: 0.65; *P* = 1.3E−5). **B.** M2S0B1 representation (Pearson’s *r*: 0.71; *P* = 8.9E−7). **C.** M1S1B1 representation (Pearson’s *r*: 0.62; *P* = 4.7E−5). **D.** M0S3B0 representation (Pearson’s *r*: 0.48; *P* = 2.8 E−3). **E.** M0S2B1 representation (Pearson’s *r*: 0.61; *P* = 5.3 E−5). **F.** M0S0B3 representation (Pearson’s *r*: 0.64; *P* = 2.1E−5).
