# Supplementary figures and images for: On the Completeness of Existing RNA Fragment Structures
Source: Genomics Proteomics Bioinformatics. 2025 Dec 18;23(6):qzaf127. doi: 10.1093/gpbjnl/qzaf127 (PMC13197129; doi:10.1093/gpbjnl/qzaf127)

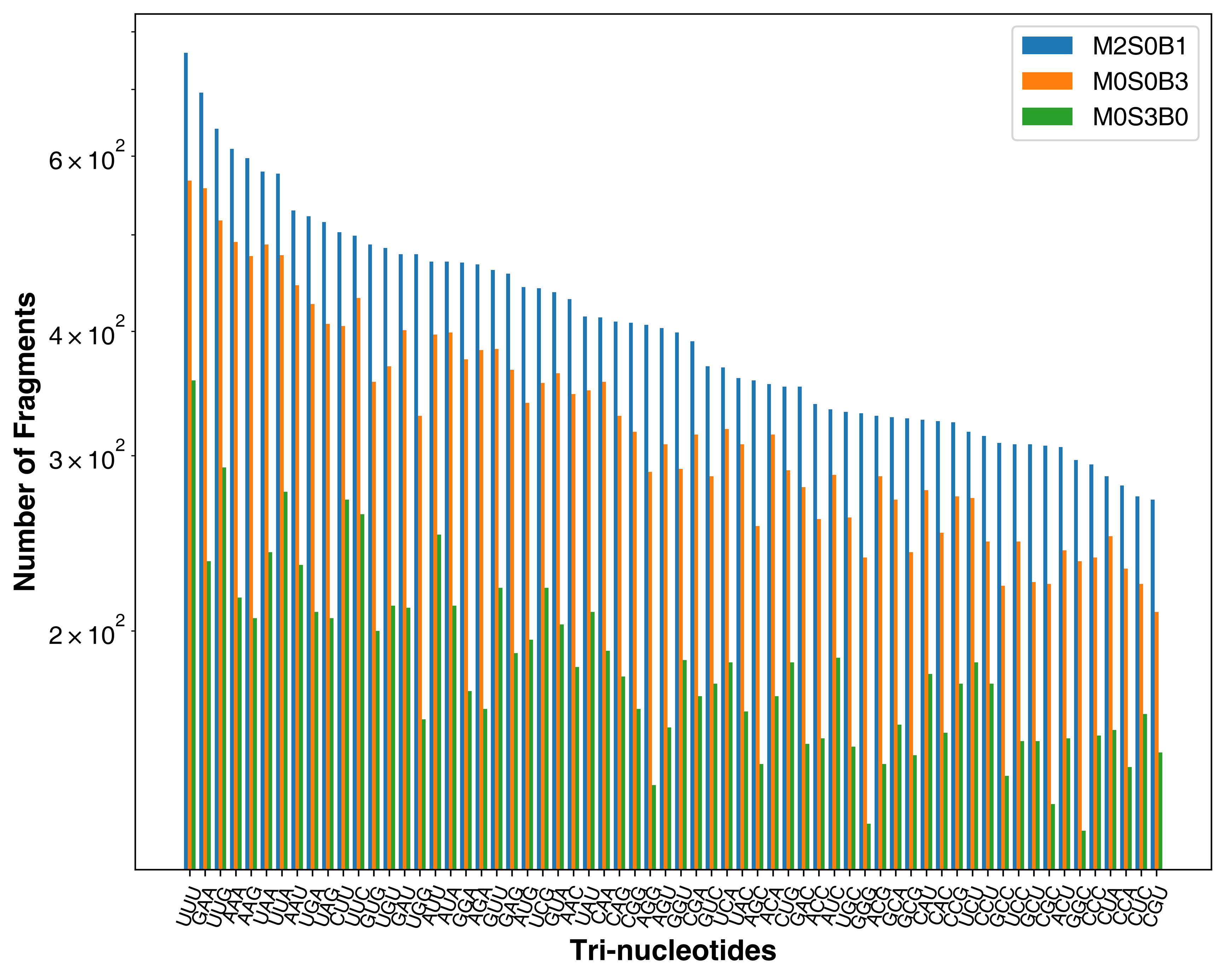

Supplement: qzaf127_Supplementary_Data [file qzaf127_supplementary_data.zip › Fig S3.tif]

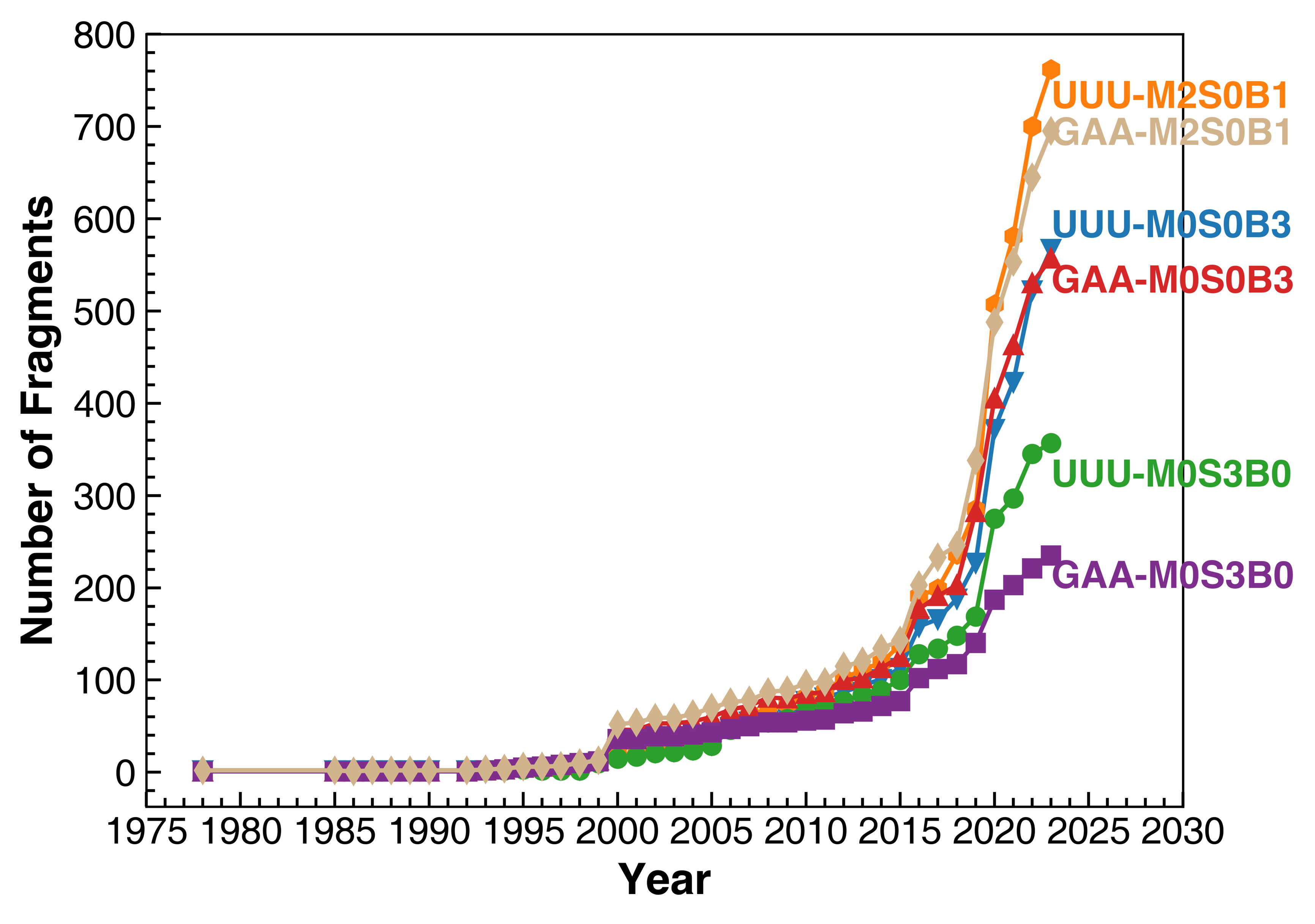

Supplement: qzaf127_Supplementary_Data [file qzaf127_supplementary_data.zip › Fig S4.tif]

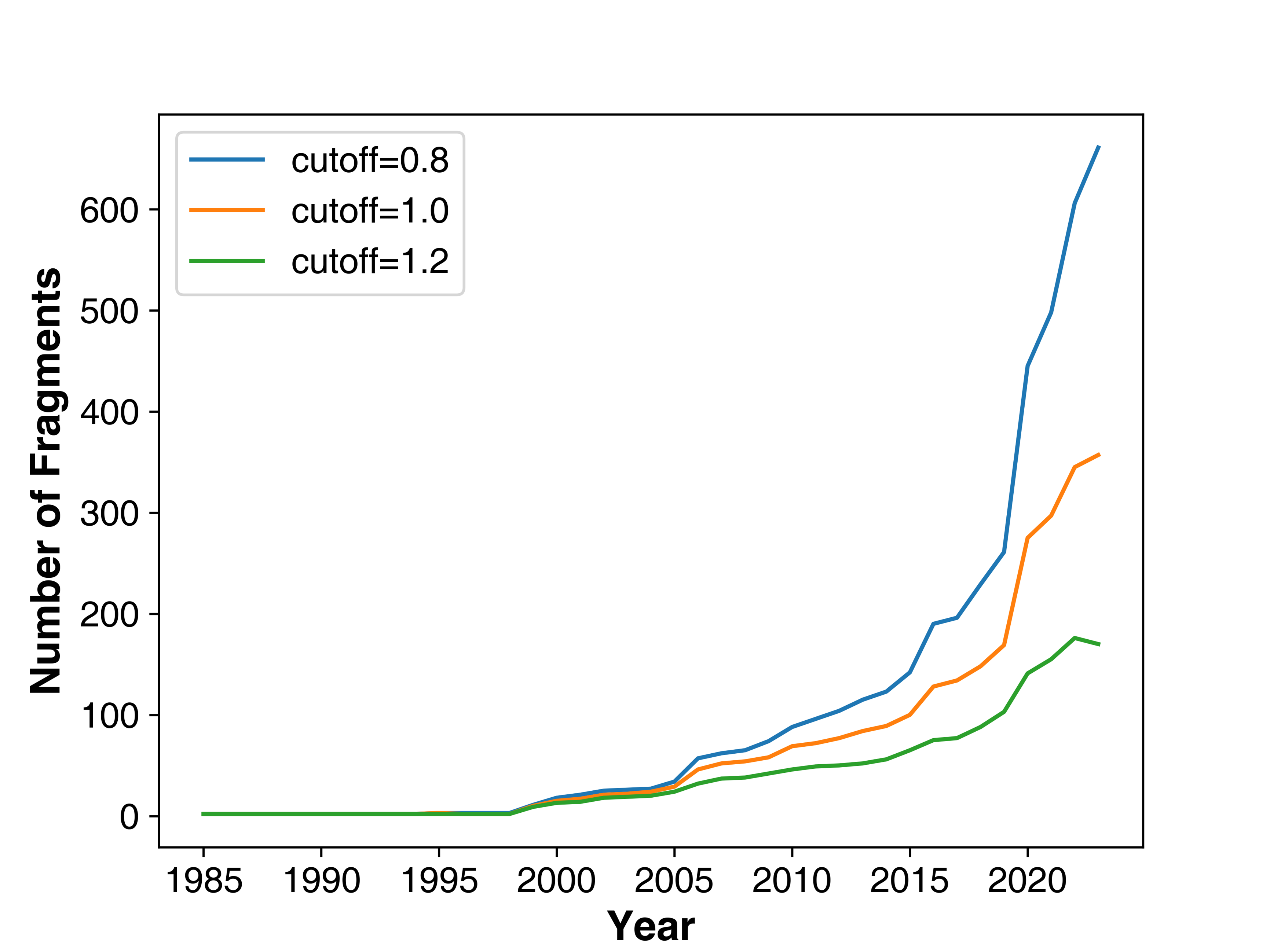

Supplement: qzaf127_Supplementary_Data [file qzaf127_supplementary_data.zip › Fig S5.tif]

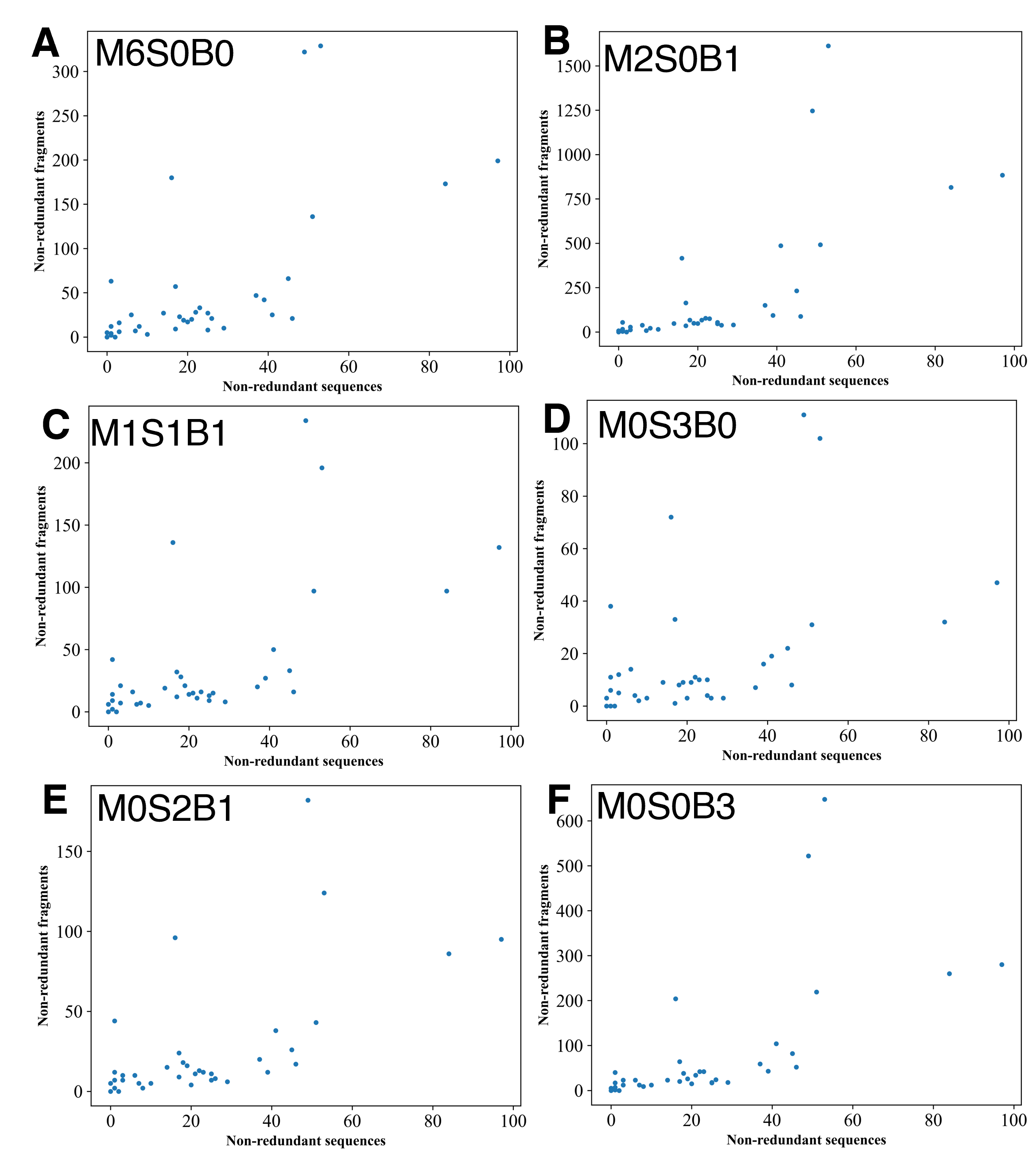

Supplement: qzaf127_Supplementary_Data [file qzaf127_supplementary_data.zip › Fig S6.tif]

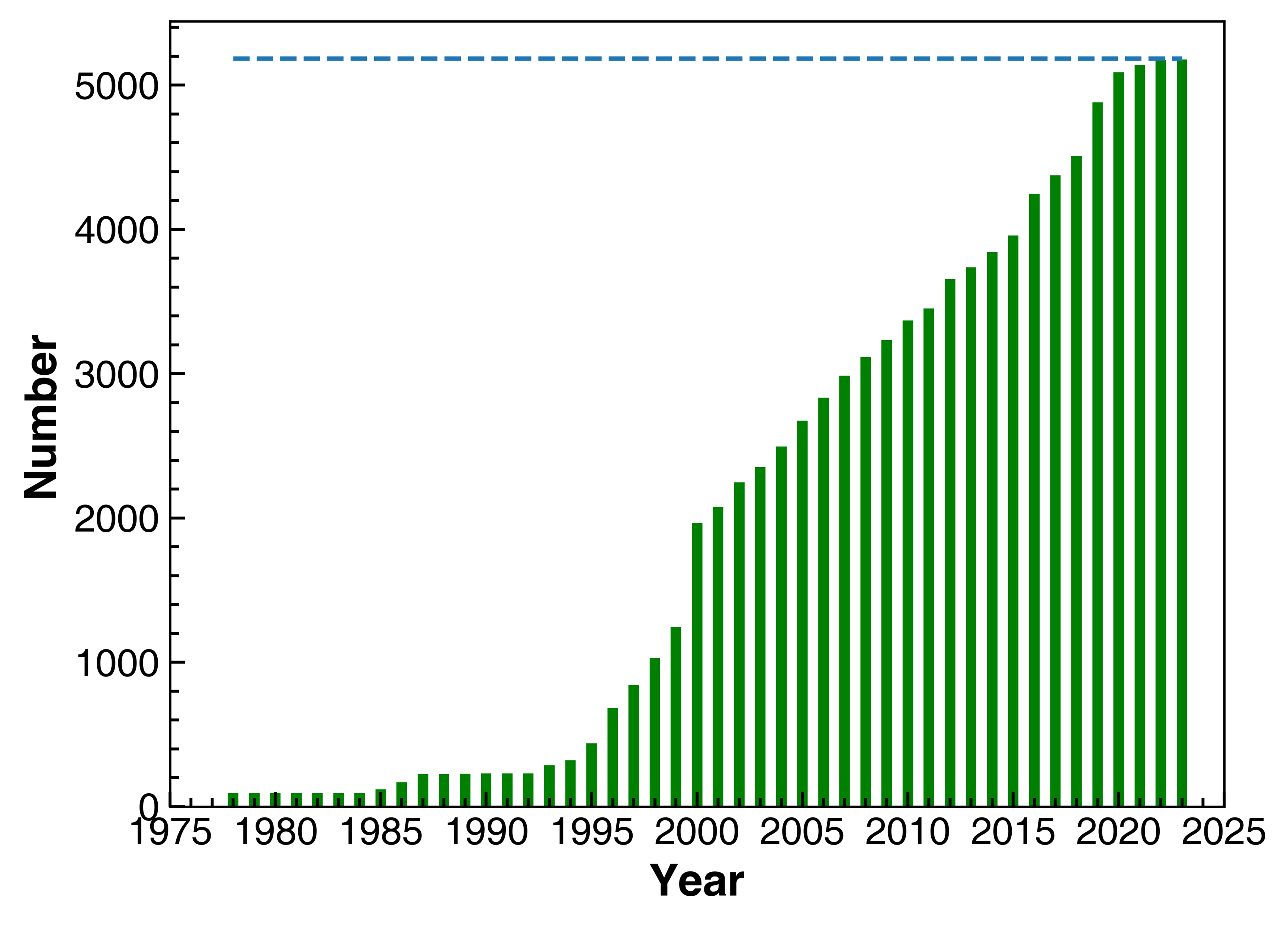

Supplement: qzaf127_Supplementary_Data [file qzaf127_supplementary_data.zip › Fig S1.tif]

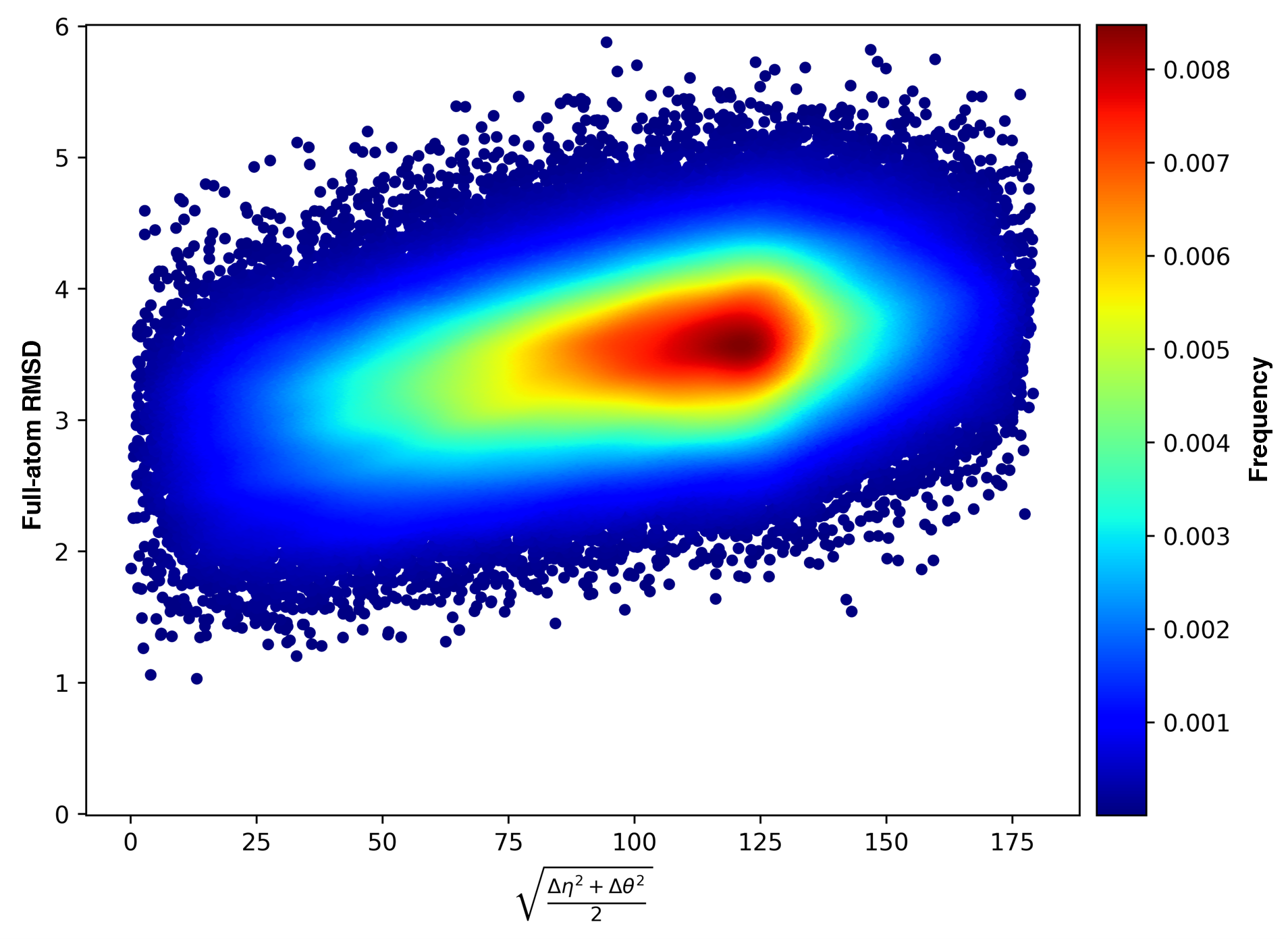

Supplement: qzaf127_Supplementary_Data [file qzaf127_supplementary_data.zip › Fig S2.tif]
